# Base editing and nanoparticle transfection of airway cell types essential for treatment of cystic fibrosis

**DOI:** 10.1172/jci.insight.198563

**Published:** 2026-05-08

**Authors:** Erin W. Kavanagh, Anya T. Joynt, Audrey R. Pion, Alice C. Eastman, Alianna I. Parr, Katherine L. Starego, Manav Jain, Sydney R. Shannon, Edwin J. Yoo, Gregory A. Newby, Stephany Y. Tzeng, Neeraj Sharma, Jordan J. Green, Garry R. Cutting

**Affiliations:** 1 Department of Genetic Medicine, Johns Hopkins University School of Medicine, Baltimore, Maryland, USA.; 2 Department of Biomedical Engineering and; 3 Institute for NanoBioTechnology, Johns Hopkins University, Baltimore, Maryland, USA.

**Keywords:** Clinical Research, Genetics, Gene therapy, Genetic diseases, Transcriptomics

## Abstract

Cystic fibrosis (CF) is a life-limiting genetic disorder caused by deleterious variants in the *CFTR* gene that results in altered mucus impairing the airway epithelia. Durable correction of these variants in airway cells remains a therapeutic challenge for about 10% of individuals unresponsive to CFTR modulators. A common disease-causing *CFTR* splice site variant, 3120+1G>A, was corrected in primary CF airway cells using base editor RNAs. Single-cell RNA sequencing revealed a remarkable increase in detectable *CFTR* transcript in most CF airway epithelial cell types resulting in notable enrichment of *CFTR*-expressing ionocytes and secretory goblet cells. Progenitor basal cell subtypes were edited, but they decreased as a fraction of total cells and *CFTR*-expressing cells compared with unedited cells. CRISPR base editors delivered by polymeric nanoparticles (PNPs) facilitated functional rescue of CFTR to clinically meaningful levels in immortalized and primary airway cells. PNPs delivered GFP-encoding RNA to progenitor airway cells in fully differentiated airway cultures. Vitronectin was a major component of the PNP corona that formed in vivo, but preincubation with vitronectin did not enhance delivery. Together, these findings validate a scalable, nonviral platform with compelling translational promise for treating CF and other respiratory diseases involving respiratory epithelial cell dysfunction.

## Abstract 2

Gene editing corrects a common disease-causing variant in cell types essential for the treatment of lung disease in persons with Cystic Fibrosis

## Graphical abstract

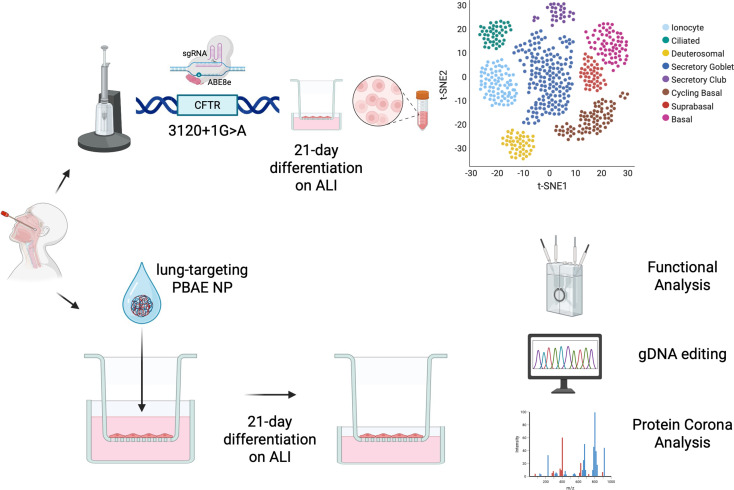

## Introduction

Current treatments for cystic fibrosis (CF) primarily focus on modulator drug therapies designed to correct malfunctioning CF transmembrane conductance regulator (CFTR) protein (1). However, these modulators are ineffective for individuals with CF carrying variants that do not synthesize CFTR protein. CRISPR/Cas9–mediated base editing can correct DNA variants to allow production of sufficient protein to achieve clinically relevant levels of CFTR function (2–8). Among these is the canonical splice variant c.2988+1G>A (legacy 3120+1G>A), the most common CF-causing variant in native Africans (9, 10). The variant ablates splicing of exon 18, leading to a frameshift, introduction of premature termination codons causing transcript instability due to mRNA degradation, and loss of protein synthesis (11). We have demonstrated that electroporation of a modified CRISPR/Cas9 adenine base editor (ABE) to primary human bronchial epithelial (HBE) cells carrying 3120+1G>A achieved 75% allelic editing resulting in about 40% WT CFTR function (2). These are encouraging results; however, successful translation to individuals affected with CF requires evidence that editing systems correct epithelial cell types where CFTR plays a key role in ion and fluid transport. In conjunction, delivery vehicles must be evaluated for efficient transfer of components that affect editing of airway epithelial cells and recovery of CFTR function.

Characterization of the transcriptome of individual cells by scRNA-seq has deeply informed our understanding of cellular composition of surface airway epithelial cells in various locations in the bronchial tree. This method enabled the discovery of ionocytes, a rare cell type that expresses the highest level of *CFTR* mRNA (12, 13) and is a key mediator of airway surface liquid (ASL) volume, pH, and ionic content (14–17). Despite this critical role, ionocytes account for only a small fraction of epithelial cells (1%–2%) in the airways of mice, ferrets, and humans. Although secretory epithelial cells individually express *CFTR* at a lower level than ionocytes, their commonness (~20%–40%) accounts for 40%–60% of *CFTR* transcripts in large and small airways (18, 19). Basal cells, the progenitor cell type that is responsible for surface epithelial cell replenishment (20), also individually express modest levels of *CFTR* mRNA, but they account for 15% to 50% of airway cells directly assayed from human lungs (18, 19). These studies indicate that correction of CFTR, particularly in the small airways where CF lung pathology is most evident, should broadly encompass most subsets of secretory and basal cells in order to maximize its therapeutic effect. In addition, the rare ionocytes should be edited as they are key mediators of ASL.

Comparison of freshly obtained healthy and CF epithelial cells reveals relatively similar cellular distributions except that CF samples have an increase in subsets of ciliated and secretory cells and a decrease in cycling basal cells (18). When *CFTR* percentage is assessed, healthy and CF individuals have similar distributions except for ionocytes; CF samples exhibit 2-fold increases in *CFTR*-positive (*CFTR^+^*) cells and overall fraction of *CFTR* expression (18). While studies of fresh samples of the airways are desirable, these are challenging to obtain and transport, especially from individuals carrying less common *CFTR* genotypes that are more likely to be selected for gene correction treatments. Expansion and differentiation of airway epithelial cells obtained from bronchial lavage or nasal turbinate brushing using Rho kinase (ROCK) inhibitors and air-liquid interface (ALI) provide a flexible solution to studying *CFTR* variants and their response to therapy. All major cell types observed in fresh samples of CF and non-CF subjects are found in cultured cells, with some differences in select subsets (18, 21, 22). Furthermore, scRNA-seq of cultured nasal cells reveals that the distribution of *CFTR*-expressing cells corresponds to the distribution in small airways, the primary site of disease in CF (21, 23). Overall, nasal and bronchial cells cultured in ALI provide a reasonable representation of the differentiation process in vivo and the cell types that should express CFTR for restoration of normal ion and fluid transport.

Of equal importance to knowing what cells need to have restored CFTR function is to be able to deliver therapeutic components to those cells. For the lungs, administration of aerosolized viral and nonviral delivery vehicles has been explored extensively, although barriers to efficient transfection of the CF lung still need to be surmounted (24, 25). Systemic delivery of nonviral vectors has shown utility for single-gene disorders requiring correction in hepatocytes (26). However, specialized formulations are required to deliver nanoparticles (NPs) to organs affected in CF such as the lungs and pancreas (21, 22). An alternative approach is to formulate nanomaterials inherently suited for lung transfection. One such class of materials are biodegradable polymeric NPs (27–29) that enable efficient delivery of reporter mRNAs to primary human airway cells in vitro and mouse lung epithelial cells in vivo (30, 31).

To determine which *CFTR*-expressing cell types are edited and whether gene correction alters cell types upon differentiation, we performed scRNA-seq of primary cells after editing of the 3120+1G>A variant. To address transfer, we tested the efficiency of polymeric NPs encapsulating base editing machinery to edit in differentiated immortalized cystic fibrosis bronchial epithelial (CFBE) cells with an integrated *CFTR* expression minigene bearing the 3120+1G>A variant. This approach was extended to primary airway cells bearing 3120+1G>A prior to ALI differentiation. The final step involved administration of polymeric NPs encapsulating GFP mRNA to fully differentiated HBE cells. Together, these studies determined which cell types undergo gene editing and generate corrected *CFTR* mRNA transcripts, as well as the effect of *CFTR* correction on respiratory cell types and the ability of polymeric NPs to deliver editing cargo to differentiating human airway cells.

## Results

### Editing with different guide RNAs results in substantial increases in number of primary airway cells with detectable CFTR transcript.

**Figure 1:**
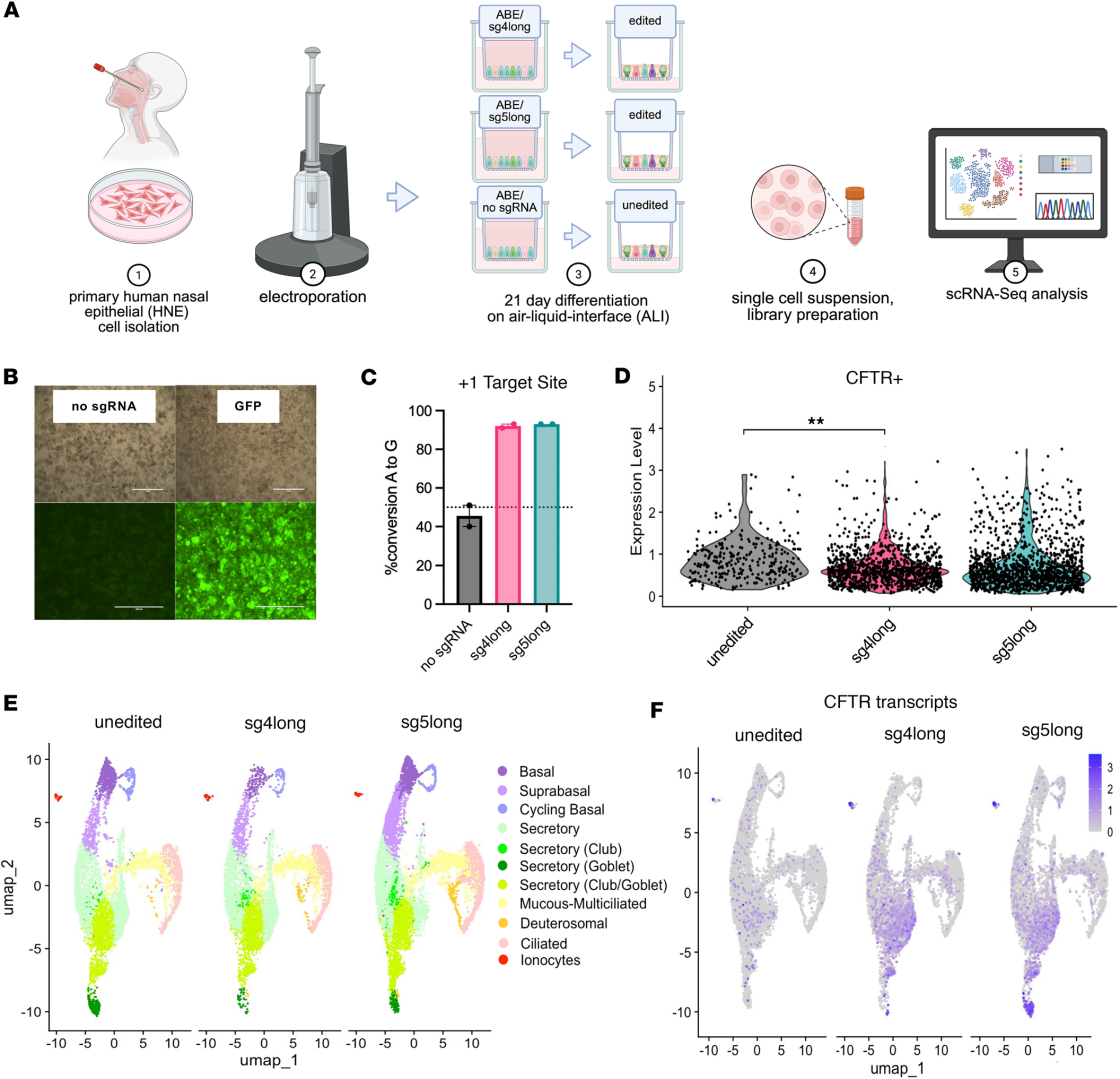
Single-cell transcriptome atlas of primary HNE cells from a CF donor bearing the variants 3120+1G>A/G480S corrected with an ABE. (**A**) Schema of primary HNE cell collection, electroporation, and differentiation period on air-liquid interface. Cells underwent electroporation and delivery of ABE8e and sgRNA (4long or 5long) before undergoing differentiation for 21 days, and then were processed for scRNA-seq. (**B**) Fluorescent microscopy images taken 24 hours after electroporation of ABE8e mRNA/no sgRNA (left) and GFP mRNA (right) with bright-field (top) or GFP (bottom). Scale bars: 1,000 μm. (**C**) Quantification of gDNA A-to-G nucleotide conversion (% G) at the 3120+1G>A target site. Dashed line represents 50% G nucleotide content to indicate contribution of G sequence from the G480S allele in *trans*. Values were determined using the Sanger sequencing deconvolution program EditR (64). Data are shown as mean ± SEM (*N* = 2). (**D**) Violin plot comparing CFTR expression levels in *CFTR^+^* cells only across edited and unedited samples. *P* value was determined by 1-way ANOVA followed by Tukey’s multiple-comparison test. **P ≤ 0.01. (**E**) UMAPs separated by edited (*N* = 4 [sg4long *N* = 2, sg5long *N* = 2]; 13,305 cells) or unedited (*N* = 2 [control]; 9,906 cells) samples. (**F**) UMAP detailing CFTR expression across all cellular subtypes.

To ensure broad and highly efficient transfection, we used electroporation to transfect primary human nasal epithelial (HNE) cells from an individual bearing 3120+1G>A (c.2988+1G>A)/G480S (c.1438G>A) with ABE8e mRNA and 1 of 2 sgRNAs (sgRNA4long or sgRNA5long) with similar editing efficiency (2). Edited and unedited cells were cultured in parallel using the same media and supplements. Single-cell RNA sequencing (scRNA-seq) was performed on cells differentiated for 21 days (Figure 1A). Transfection with RNA encoding green fluorescent protein (GFP) was used as a proxy to evaluate best electroporation conditions (Figure 1B). DNA editing of the target nucleotide in HNE cells was efficient, with 85% A>G conversion with sgRNA4long (sg4long) and 88% with sgRNA5long (sg5long) (Figure 1C). Conversion was calculated after taking into account the heterozygous status at the target site due to the presence of the other *CFTR* allele (G480S) and was comparable to previous reports (2). The average *CFTR* expression in cells having detectable *CFTR* transcript (i.e., *CFTR^+^* cells) was similar between unedited (2.07) and edited cells (sg4long 1.69; sg5long 2.04) (Figure 1D). However, the number of cells with detectable *CFTR* transcript increased 5.2-fold between unedited (0.06) and sg4long (0.31) and 6.9-fold between unedited and sg5long (0.41) (Supplemental Figure 1; supplemental material available online with this article; https://doi.org/10.1172/jci.insight.198563DS1).

An integrated uniform manifold approximation and projection (UMAP) assembled using all 23,211 cells revealed 11 distinct cellular subsets, including basal, secretory club and goblet, ciliated, and ionocytes (Supplemental Figure 2), using marker genes for each subset characterized in prior publications (Supplemental Figures 3 and 4) (13, 19, 21, 32). Unedited and edited CF cells had higher proportions of secretory and ciliated cells but lower fractions of basal cell types than nasal cultures derived from healthy (WT) individuals (Supplemental Figure 5A), as reported by others (18, 21). All major subclusters were present in projections of unedited (9,906 cells), edited with sg4long (6,231 cells), and edited with sg5long (7,074 cells) (Figure 1E). At this level of resolution, ionocytes were differentiated from tuft/brush cells (Supplemental Figure 6), the proposed progenitor of ionocytes (14, 33). The UMAPs revealed that editing with either guide RNA resulted in similar substantial increases in number and similar distribution of cells with detectable *CFTR* transcript (Figure 1F). Transcription of the *CFTR* gene bearing G480S accounted for the *CFTR* mRNA in the unedited cells.

### Editing increases detectable CFTR expression in ionocytes and secretory cells, resulting in CFTR function increases to clinically relevant levels.

**Figure 2:**
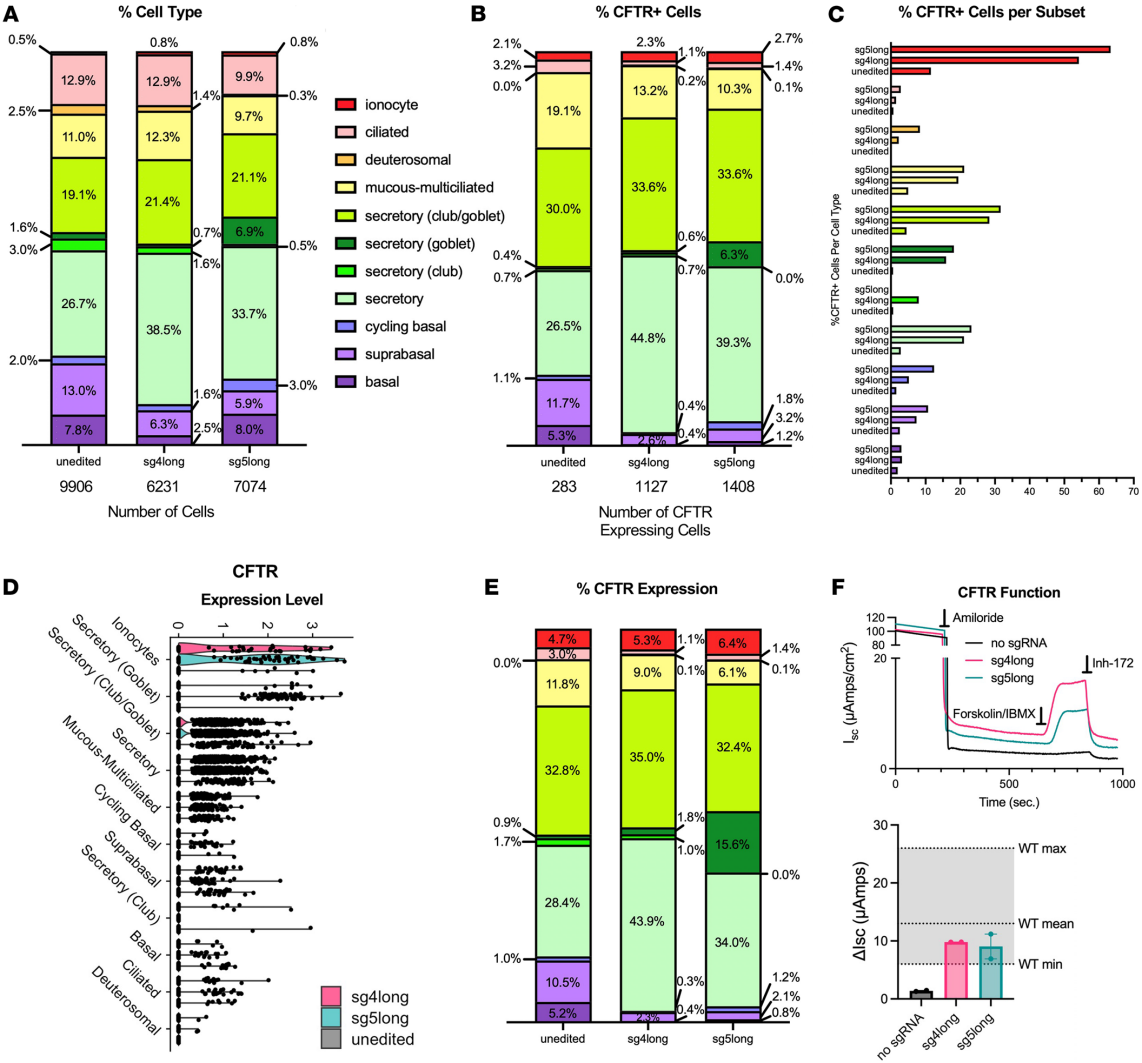
Graphical representations of cell type distribution and CFTR upregulation, distribution, and expression between primary HNE samples unedited (no sgRNA) or edited with sg4long or sg5long. (**A**) Cellular composition across cellular subsets. (**B**) Percentage *CFTR^+^* cells across cellular subsets. (**C**) Percentage *CFTR^+^* cells per cellular subset. (**D**) Violin plot comparing CFTR expression levels across cellular subsets. (**E**) CFTR expression across cellular subsets, *CFTR^+^* cells only. (**F**) Top: Representative short-circuit current tracings of CFTR functional recovery in primary HNE cells bearing the 3120+1G>A/G480S variants after electroporation of ABE/no sgRNA (unedited; black), ABE/sg4long (pink), and ABE/sg5long (green). Each representative tracing was aligned according to the same baseline after addition of amiloride. Bottom: Quantified CFTR functional recovery as ΔI_sc_ (μA/cm^2^). Gray shading indicates ranges of values observed in WT/WT HNE cells as previously reported (35). Data are shown as mean ± SEM (2 biological replicates from 1 transfection).

To assess editing efficiency in cells endogenously expressing *CFTR* (*CFTR^+^*), we probed individual cellular subsets. Most subsets were found to have similar proportions between edited and unedited samples, excluding the basal and secretory populations, where editing with either guide RNA resulted in a lower percentage of basal and a higher percentage of secretory subsets (Figure 2A and Supplemental Figure 7A). Both edited samples revealed a different *CFTR*-expressing cellular makeup from unedited cells. Notably, among all *CFTR^+^* cells, the fraction attributable to ionocytes increased by approximately 20% in edited samples (Figure 2B and Supplemental Figure 7B) owing to a nearly 5-fold increase in ionocytes with detectable *CFTR* mRNA (Figure 2C and Supplemental Figure 7C). Notably, expression level per ionocyte did not increase in the cells edited with either guide RNA (Figure 1D). However, the greater number of ionocytes with detectable levels of *CFTR* resulted in an increase in total percentage *CFTR* expression attributable to ionocytes with each guide RNA (Figure 2E) that translated to an approximately 26% increase (from 4.7% to 5.8%) when results from each guide RNA were combined (Supplemental Figure 7C).

Generally, editing was associated with an increase in secretory populations and a decrease in progenitor and ciliated. However, there were exceptions in subtypes; cycling basal cells had a 0.3% and 0.1% increase in cell type and *CFTR^+^* cell composition, respectively. Inversely, secretory club cells had a 2.8% and 0.4% decrease in cell type and *CFTR^+^* cell composition, respectively (Supplemental Figure 7, A and B). Most cell types saw dramatic increases in the fraction of cells with detectable *CFTR* (Figure 2C and Supplemental Figure 7C), while *CFTR* expression level in each cell type remained about the same when unedited and edited samples were compared (Figure 2D). As expected, the shifts in cell populations resulted in similar shifts in the percentage *CFTR* expression; secretory cells accounted for a larger fraction, while progenitor and ciliated cell types accounted for a reduced portion (Figure 2E and Supplemental Figure 7D). The 2 sgRNAs exhibited modest differences. Goblet cells, like ionocytes, had high levels of *CFTR* expression per cell. Editing with sg5long increased the number of goblet cells with detectable *CFTR* mRNA, resulting in this cell type accounting for approximately 16% of *CFTR* expression after editing, but there was only a modest increase of 0.9% with sg4long (Figure 2E). Inversely, the fraction of *CFTR* expression attributable to secretory cells with sg4long increased from about 28% to about 44% after editing, while the fraction increased to just ~34% with sg5long (Figure 2E and Supplemental Figure 7D). Both samples of edited cells had lower fractions of basal cells, constituting a small fraction of *CFTR^+^* cells after editing. Individual values for percentage *CFTR^+^* cells from each subset, *CFTR* expression levels, percentage cellular makeup, and percentage *CFTR^+^* cellular makeup are shown in Supplemental Figure 8, A–D.

To determine whether editing shifted cell types toward a WT (i.e., non-CF) distribution, we compared results with a previously analyzed scRNA-seq dataset from healthy HNE cells cultured in the same manner and at the same time point of differentiation. Secretory cells accounted for larger fractions of cells with detectable *CFTR* expression and overall percentage *CFTR* expression in the CF samples (Supplemental Figure 5, B and C), which aligns with previous reports (18, 21). Because of the method of data analysis in the WT samples, we could not define as many secretory cell subsets as our prior analysis. However, proportions of cells remained directly comparable since both samples underwent processing at 21 days on ALI culture. Notably, ionocytes were increased and basal cell types were decreased in *CFTR^+^* populations and in overall percentage *CFTR* expression in CF samples. Editing increased the ionocyte fraction but further reduced the fraction of basal cells among *CFTR^+^* cells and as percentage of overall *CFTR* expression. Others have reported similar results when full-length WT *CFTR* is expressed as a transgene in CF airway cells (34).

CFTR functional recovery was assessed by short-circuit measurement in Ussing chambers as previously described (35). After addition of amiloride to inhibit the epithelial Na^+^ channel (ENaC), CFTR activity was stimulated using a combination of forskolin and 3-isobutyl-1-methylxanthine (IBMX), and CFTR chloride transport was measured as the change in current in response to CFTR-specific inhibitor, inh-172 (Figure 2F). The increase in short-circuit current (ΔI_sc_) in primary cells edited with either guide RNA (sg4long: 9.8 ± 0.01 μA/cm^2^; sg5long: 9.1 ± 2.1 μA/cm^2^) was substantially higher than that in unedited cells (1.4 ± 0.1 μA/cm^2^) and reached ranges previously reported in WT HNE cells (Figure 2F) (35). The substantial increase in current in edited cells is consistent with the increased number of airway epithelial cells with detectable *CFTR* transcript in most cell types, particularly ionocytes and secretory cells.

### Polymeric NPs delivering ABE/sgRNA to immortalized CFBE cells bearing the 3120+1G>A variant achieve clinically relevant CFTR functional recovery.

**Figure 3:**
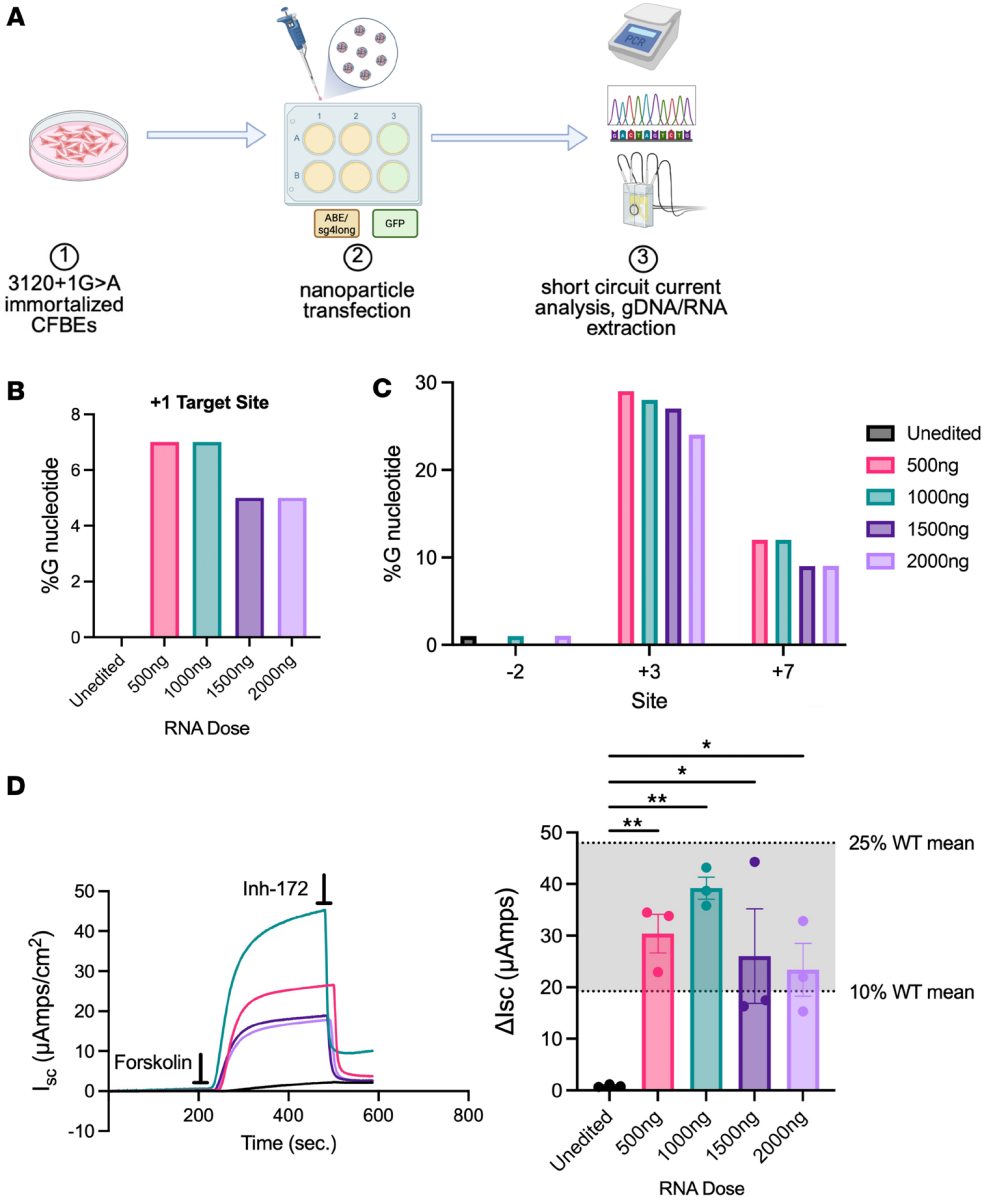
PBAE-E63 nanoparticles delivering ABE8e/sg4long correct 3120+1G>A variant and restore CFTR function in isogenic CFBE cells. (**A**) Schema of immortalized CFBE cells bearing the 3120+1G>A variant transfected with PBAE-E63 encapsulating either ABE8e/sg4long or GFP mRNA before analysis by short-circuit current analysis and gDNA and RNA extraction. (**B** and **C**) Quantification of gDNA nucleotide conversion at the 3120+1G>A (+1) target site (**B**), as well as potential bystander adenine edits (–2, +3, +7) after delivery of ABE and sg4long to CFBE cells stably expressing EMG_i14-i18 bearing the 3120+1G>A variant (**C**). Values were determined by linear/PCR sequencing using Oxford Nanopore technology. (**D**) Left: Representative short-circuit current tracings of CFTR functional recovery in isogenic CFBE cells bearing the 3120+1G>A variant of interest after delivery of GFP (unedited; black), 500 ng (pink), 1,000 ng (green), 1,500 ng (dark purple), or 2,000 ng (light purple) with PBAE-E63 encapsulating ABE8e/sg4long. Horizontal bars indicate timing of application of each compound, which was sequential. Right: Quantified CFTR functional recovery as ΔI_sc_ (μA/cm^2^) across 4 RNA doses. Gray shading indicates ranges of values observed in WT CFBE cells stably expressing EMG_i14-i18 as previously reported (2). Data are shown as mean ± SEM (3 biological replicates from 1 transfection). *P* values were determined by 1-way ANOVA followed by Dunnett’s multiple-comparison test. *P ≤ 0.05, **P ≤ 0.01.

Once we had demonstrated that *CFTR* expression was recovered in the cell types essential for CF treatment, we tested the efficiency of editing upon delivery of the base editor RNA using polymeric NPs (PNPs), a clinically viable approach as opposed to electroporation. Our first step was to assess delivery and editing efficiency after PNP delivery to immortalized CFBE cells bearing the integrated *CFTR* expression minigene with 3120+1G>A (Figure 3A). PNPs encapsulating NRCH-ABE8e mRNA and the sg4long RNA were tested over a range of RNA concentrations per PNP formulation (5, 7.5, and 10 μg RNA) and PNP volumes (1, 2, 5, 10, and 20 μL), resulting in delivered RNA ranging from 50 to 1,000 μg. Sg4long was chosen because of its ability to achieve higher functional recovery of CFTR, likely due to reduced bystander editing (2). The highest DNA editing efficiency (7%) was observed at the lower doses (500 ng and 1,000 ng), while 5% efficiency was noted at the 2 highest doses (1,500 ng and 2,000 ng) (Figure 3B). Furthermore, the 1,000 ng dose had slightly reduced editing at the +3 bystander site (Figure 3C), which is known from previous work to cause minimal (~20%) exon 18 skipping, resulting in the same aberrant splice isoform generated by 3120+1G>A (2). CFTR function assessed by short-circuit current after forskolin activation and inh-172 inhibition (Figure 3D) revealed that unedited cells showed minimal current (0.9 ± 0.1 μA/cm^2^), whereas PNP-transfected cells with ABE8e mRNA and sgRNA generated a response between 23 ± 5 and 39 ± 2 μA/cm^2^, all within the 10%–25% WT therapeutic range (Figure 3D).

The 1,000 ng dose led to the highest recovery of function, equating to 20% of the function observed in WT CFBE cells, despite the same level of editing at the target site as 500 ng. Earlier studies with GFP mRNA delivery reveal a consistent 80% transfection across all doses while maintaining a viability of greater than 90%; however, mean fluorescence intensity does decrease as dose increases (Supplemental Figure 9). This indicates there are other factors at play when base editor machinery is delivered. Observations with CFBE cells noted that delivery with the PNPs was able to correct 3120+1 to clinically relevant ranges with relatively low RNA dosing and low rates of editing.

### PBAE PNPs delivering ABE/sgRNA to primary HBE cells bearing the 3120+1G>A variant achieve up to 50% WT CFTR functional recovery.

**Figure 4:**
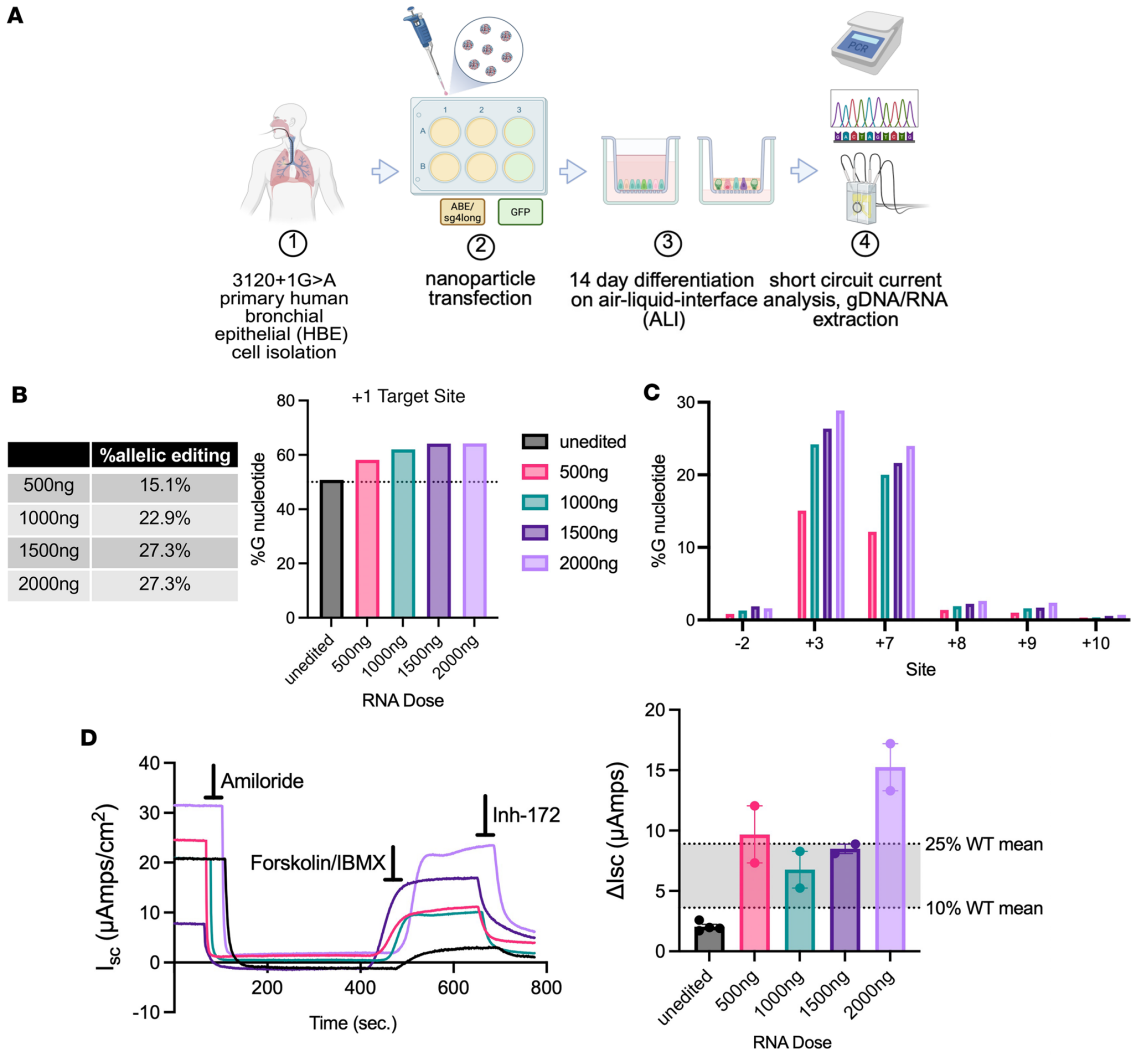
PBAE-E63 nanoparticles delivering ABE8e/sg4long to primary HBE cells bearing the 3120+1G>A/F508del variants correct 3120+1G>A and restore CFTR function. (**A**) Schema of primary HBE isolation, NP transfection, and differentiation period on air-liquid interface. Cells were transfected with PBAE-E63 encapsulating either ABE8e/sg4long or GFP mRNA before undergoing differentiation for 14 days, then short-circuit current analysis and gDNA and RNA extraction. (**B**) Left: Levels of allelic editing efficiency under each condition, calculated as: % allelic editing = [(% G_edited_ – % G_unedited_) / % A_unedited_] × 100 (5). Right: Quantification of gDNA nucleotide conversion (% G) at the 3120+1G>A target site (+1). Dashed line represents 50% G nucleotide content to indicate contribution of G sequence from the F508del allele in *trans*. (**C**) A-to-G conversion rate at bystander adenine sites (–2, +3, +7, +8, +9, +10). Values were determined using MiSeq and CRISPResso (66). (**D**) Left: Representative short-circuit current tracings of CFTR functional recovery in primary HBE cells bearing the 3120+1G>A/F508del variants after delivery of GFP (unedited; black), 500 ng (pink), 1,000 ng (green), 1,500 ng (dark purple), or 2,000 ng (light purple) with PBAE-E63 encapsulating ABE8e/sg4long. Horizontal bars indicate timing of application of each compound, which was sequential. Right: Quantified CFTR functional recovery as ΔI_sc_ (μA/cm^2^) across 4 RNA doses. Gray shading indicates ranges of values observed in WT/WT HBE cells as previously reported (2). Data are shown as mean ± SEM (2 biological replicates from 1 transfection).

Our next step was to assess editing efficiency of PNPs delivered to primary HBE cells bearing the 3120+1G>A/F508del variants. NPs encapsulating NRCH-ABE8e mRNA and sg4long were formulated at 4 different doses (500 ng, 1,000 ng, 1,500 ng, 2,000 ng) and delivered to cells adhered to a 6-well dish. Forty-eight hours after transfection, cells were moved to filters, where they were allowed to form a confluent monolayer of cells (about 2–3 days), then transitioned to ALI culture to allow for differentiation (Figure 4A). Delivery of ABE8e and sgRNA achieved 7.4% correction at 500 ng, 11.3% correction at 1,000 ng, 13.4% correction at 1,500 ng, and 13.5% correction at 2,000 ng doses (Figure 4B). After subtraction of the contribution from the F508del allele in *trans* (dashed line, Figure 4B) (5), allelic editing calculated at the 4 RNA concentrations was 15.1%, 22.9%, 27.3%, and 27.3%. Bystander editing was noted at the –2, +1, +3, and +7 sites as previously described (2), along with editing at the +8 and +9 sites (Figure 4C).

CFTR function was measured by short-circuit current on fully differentiated HBE cells (14 days on ALI culture). As noted above, CFTR chloride transport was defined as the change in current in response to CFTR-specific inhibitor, inh-172 (ΔI_sc_) (Figure 4D). Unedited cells showed minimal current (2.0 ± 0.2 μA/cm^2^), whereas cells transfected with PNPs encapsulating ABE8e mRNA and sgRNA generated a response between 7 ± 2 and 15 ± 2 μA/cm^2^, with the highest dose equating to 50% of the function observed in WT/WT primary HBE cells (Figure 4D) (2). This level of function is directly comparable to results achieved by electroporation of the same cargoes in previous studies (2), and is consistent with our observation above that editing substantially increases the number of *CFTR*-expressing ionocytes and secretory cells, the primary mediators of chloride transport.

### Vitronectin is the most abundant protein in the protein corona of PBAE-E63 PNP.

**Figure 5:**
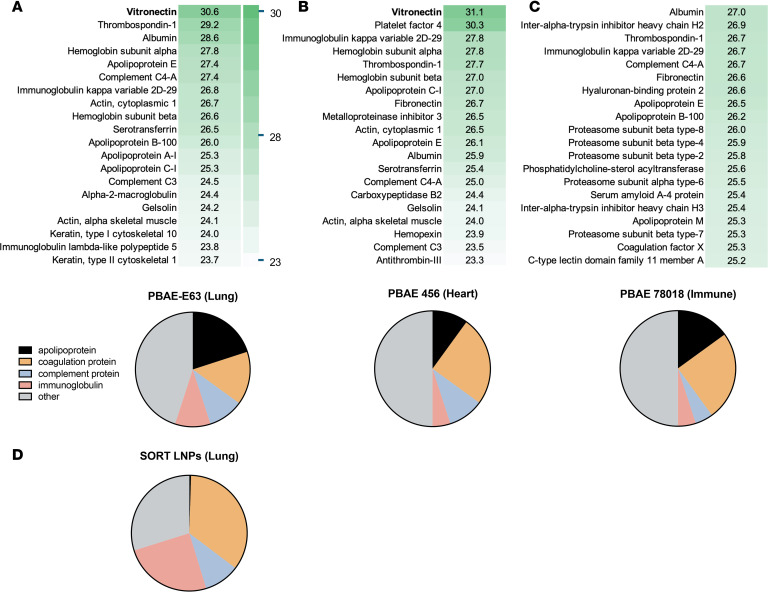
Mass spectrometry proteomics data detailing PBAE nanoparticle protein corona compositions. (**A**–**C**) Top: Heatmap depicting the top 20 most abundant proteins in lung-targeting PBAE-E63 (**A**), heart-targeting PBAE-456 (**B**), and immune cell–targeting PBAE-78018 (**C**). Bottom: Top 20 proteins classified into physiological categories. (**D**) Top 20 proteins for lung SORT-LNP protein corona from Supplementary Figure 14A in Sun et al. (4) classified into physiological categories.

We have previously shown that PNP PBAE-E63 can transfect airway epithelial cells after systemic administration to mice (30). Previous literature has suggested that the protein coating of NPs can enhance delivery to specific organs following systemic delivery (4). To investigate the constituents of protein coronas, we performed mass spectrometry on PNPs incubated in mouse blood serum. Vitronectin was identified as the protein of highest abundance in PBAE-E63 (Figure 5A), as well as cardiac-targeting PBAE-456 (Figure 5B). In comparison, noncardiothoracic-targeting PBAE-78018 designed for immune cell transfection does not contain vitronectin among the top 20 proteins (Figure 5C). While vitronectin was detected as the most abundant protein for PBAE-E63 (30.6%), levels did not differ substantially from those of other proteins detected in the coronas. This result is in contrast to the results of Sun et al. (4), who found that vitronectin was uniquely abundant (~50%) in the corona of lipid NPs (LNPs). Additionally, the top 20 protein composition of lung-targeting PBAE-E63 PNPs differed from that of selective organ targeting (SORT) LNPs targeting the lungs (Figure 5D). The PBAEs had markedly more apolipoproteins present and fewer coagulation and immunoglobulin proteins. The difference in overall top 20 protein composition between PBAE PNPs and LNPs also emphasizes the importance of overall protein corona composition as an important biomarker for potential organ and cell-specific targeting.

### PNPs can transfect fully differentiated primary HBE 3120+1G>A/F508del cells including basal cell populations.

**Figure 6:**
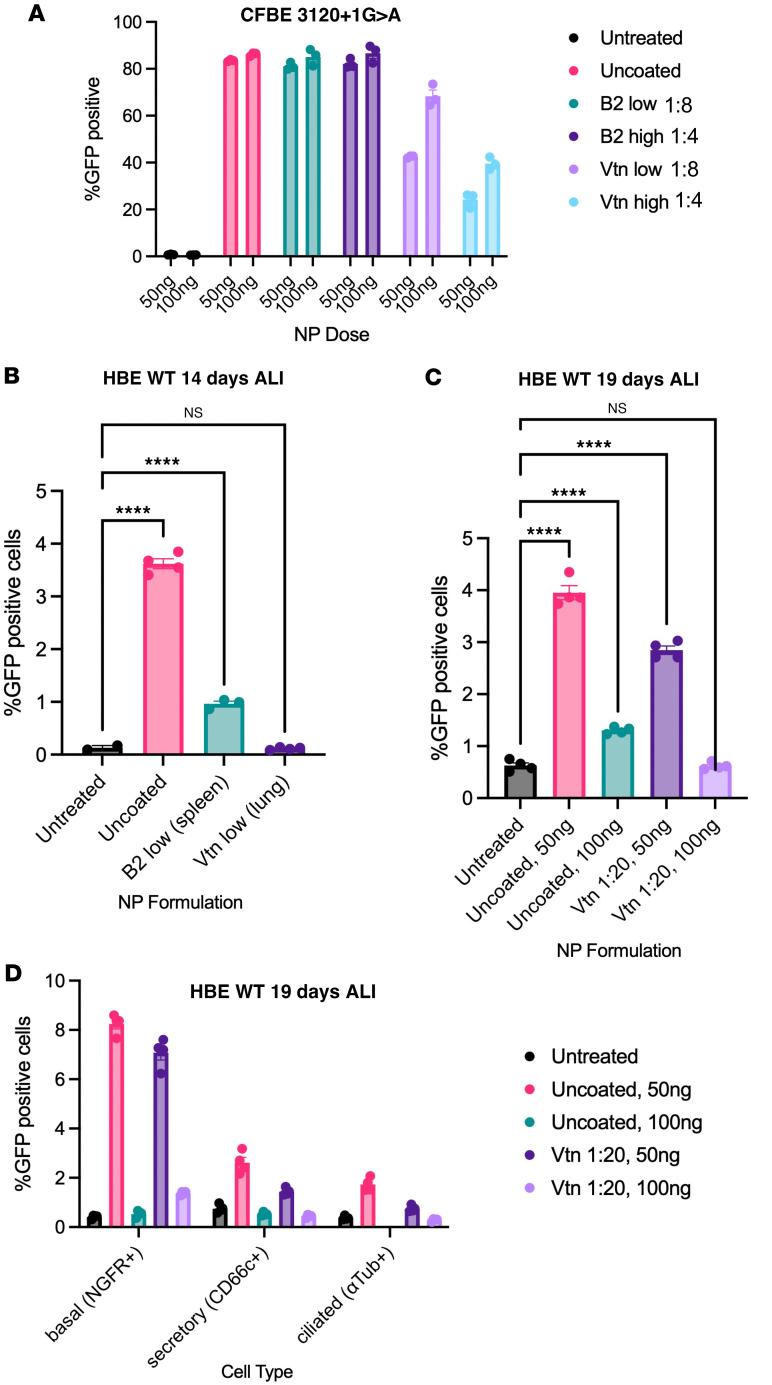
Protein-coated nanoparticle transfection with PBAE-E63, β2-glycoprotein I, and vitronectin. (**A**) Flow cytometry results of percentage GFP^+^ immortalized CFBE cells bearing the 3120+1G>A variant at 2 doses (50 ng and 100 ng) across 4 treatment conditions (untreated, uncoated, β_2_-glycoprotein I [B2] coated, and vitronectin [Vtn] coated) and 2 protein-to-PBAE coating ratios (low = 1:8, high = 1:4). Data are shown as mean ± SEM (*N* = 3). (**B**) Percentage GFP^+^ cells from flow cytometry on 14-day-differentiated HBE WT cells at 1:20 protein-to-PBAE ratio. Data are shown as mean ± SEM (*N* ≥ 2). (**C**) Percentage GFP^+^ cells from flow cytometry on 19-day-differentiated HBE WT cells at 1:20 protein-to-PBAE ratio. (**D**) Percentage GFP^+^ cells in lung cell populations (basal, secretory, ciliated) across the four NP formulation groups. Data are shown as mean ± SEM (*N* = 4). *P* values were determined by 1-way ANOVA followed by Dunnett’s multiple-comparison test. ****P ≤ 0.0001; NS, *P* > 0.05.

We next tested whether PNPs transfect fully differentiated human primary airway cells in culture and whether vitronectin coating could enhance the process. PNPs encapsulating GFP mRNA coated at various ratios with either spleen-targeting β_2_-glycoprotein I (B2) as a negative control or lung-targeting vitronectin (Vtn) were used to transfect immortalized and differentiated primary HBE cells. The highest protein-to-polymer ratio was based on suggestions in previous literature that this improved uptake (4). Flow cytometry revealed that transfection rates of immortalized CFBE cells were similar (80%–85%) when different doses (50 ng, 100 ng) of uncoated or B2-coated (low and high) PNPs were used (Figure 6A). Uncoated transfection levels were comparable to previously published results with this PNP formulation (30). In contrast, vitronectin-coated PNPs had an overall decrease in transfection, attaining a maximum of 65%.

Differentiated primary cells remain difficult to transfect. Given that the lower protein-to-polymer ratio demonstrated better transfection rates in immortalized airway cells, uncoated and coated PNPs were added to primary WT HBE cells differentiated on ALI for 14 days. Flow cytometry revealed that uncoated PNPs demonstrated the highest transfection rate of 3.6% in differentiated primary HBE cells, with B2-coated NPs demonstrating 1.0%, and vitronectin-coated NPs exhibited no transfection (Figure 6B). Because of dynamic light scattering evidence that vitronectin-coated NPs were aggregating at 1:4 and 1:8 ratios, the protein-to-polymer ratio was lowered to 1:20, and NPs were delivered to primary HBE WT cells differentiated for 19 days on ALI. Cells were antibody-stained to elucidate the 3 major lung cell populations (ciliated, secretory, basal; Supplemental Table 1), and GFP expression was measured by flow cytometry (Supplemental Figures 10 and 11). Uncoated and vitronectin-coated PBAE-E63 PNPs at the 50 ng dose had overall transfection rates of 4% and 3%, respectively, whereas higher doses of NPs were less effective (Figure 6C). A similar pattern was seen when sorted by cell types. Notably, basal cells achieved the highest rates of transfection of 8% and 7%, respectively, with secretory and ciliated cells having lower levels of transfection (Figure 6D). These results indicate that differentiated airway cells including basal cells can be transfected with PNPs, but rates are sensitive to dose and vitronectin-to-polymer ratio.

## Discussion

Gene editing provides an opportunity to cure genetic diseases by correcting deleterious DNA variants in situ. In this study, we have addressed several gaps in transitioning gene editing for cystic fibrosis to clinical trials: are cell types essential to pathophysiology corrected; does correction affect cell type distribution; and can synthetic vectors such as nanoparticles deliver cargo to essential cell types? As these are preclinical studies, we used well-characterized primary and immortalized human cells in culture to provide a reasonable approximation of differentiated epithelia lining the small airways (19), a major site of disease in CF (36–38). ScRNA-seq revealed successful correction across a broad range of epithelial cells, including those intimately involved in CFTR-dependent ion transport. We show that progenitor basal cells were edited and successfully transfected in nondifferentiated and differentiated states using PNPs.

To our knowledge, this study provides the first single-cell transcriptomic analysis of primary human airway cells after base editing to correct *CFTR*. Correction of the 3120+1G>A variant led to a striking 4- to 7-fold increase in cells with detectable *CFTR* expression, far exceeding the expected 2-fold maximum increase due to the monoallelic repair. This disproportionate upregulation occurred across all differentiated epithelial cell types, particularly ionocytes and secretory cells, that are crucial for CFTR-mediated ion transport. CFTR chloride transport increased 6- to 7-fold in edited cells, reaching clinically relevant ranges. This functional improvement closely correlated with the increase in overall *CFTR* mRNA expression, consistent with our previous findings that *CFTR* mRNA levels and CFTR chloride transport are linearly correlated in isogenic cell lines (39). Notably, CFTR function exceeded 10% of WT levels, a threshold generally recognized as sufficient to ameliorate life-limiting lung disease (40–42).

The increase in *CFTR*-expressing cells was consistent across 2 biological replicates using 2 independent guide RNA designs. Small differences in cellular patterns between the sgRNA designs might be attributed to a technical rather than biological difference given the greater experimental variability we observed with the sg4 compared with sg5 guide RNA (Supplemental Figure 8). However, it is possible that chromatin structure encompassing CFTR might differ among cell types, in which case guide RNAs might have different binding efficiencies depending on cell type. Technical limitations of scRNA-seq may account for the increase in detectable *CFTR*-expressing cells (43, 44). Indeed, cell types with the highest expression per cell of *CFTR* in unedited samples, such as ionocytes and goblet cells, showed the greatest increase in the number of cells with detectable *CFTR* transcript after editing. There are also several possible biological explanations for this disproportionate increase. One is that the promoter and/or enhancer elements driving expression of *CFTR* bearing the 3120+1G>A allele are considerably more potent than those in the accompanying gene bearing G480S. However, the average *CFTR* expression level per *CFTR^+^* cell was not higher in the edited compared with unedited cells. Another explanation is that allelic correction increased the growth rate of *CFTR*-expressing cells, which paralleled the overall increase in *CFTR^+^* cells. However, we did not observe substantial differences in growth rates between edited and unedited cells (43, 44). In sum, our results indicate that gene correction may have a disproportionately large impact on overall *CFTR* expression, potentially enhancing functional recovery beyond what would be predicted for a single allelic correction, though the mechanism of this observation remains unknown.

We expected that correction of *CFTR* would shift the developmental pattern and cellular composition of differentiated cultures, possibly to that observed in WT individuals. Following editing, we observed a modest change in the relative proportions of major cell types, with an increase in ionocytes and secretory cells and decrease in basal cells. Notably, overall nasal cell distributions in edited and unedited CF cells were more similar to each other than to the distribution of nasal cells derived from healthy individuals. These findings align with two recent studies that reported modest changes in bronchial epithelial cell distributions after transduction of basal cells with lentivirus (34) or adeno-associated viruses integrating *CFTR* cDNA (45). Although nasal epithelial cell culture models differ from freshly acquired tissues (23, 46), they still effectively recapitulate the primary role of secretory cells in overall *CFTR* expression (19), as well as the high per-cell *CFTR* expression in ionocytes and goblet cells (12, 13). Thus, while recovery of *CFTR* function in airway cells from individuals with CF does not recapitulate a WT pattern, it does not drastically distort the essential cellular populations needed for a functional differentiated epithelium.

From a therapeutic perspective, the greatest increase in *CFTR* expression after gene editing occurred in ionocytes, with the highest overall increase in secretory cells — two populations that have key roles in ion and fluid transport in airway epithelia (15–17). The increased number of airway epithelial cells with detectable *CFTR* transcript in most cell types, particularly ionocytes and secretory cells, resulted in a substantial increase in current in edited cells to levels needed for a therapeutic response. In genetically engineered ferrets, recovery of CFTR expression in nasal and tracheal epithelia has a dramatic effect on ionocyte regeneration (47), while modulator-augmented CFTR function in human nasal airways normalizes epithelial differentiation (48). Together, these observations suggest that recovery of CFTR function positively affects differentiation of key cell types that will enable a clinically meaningful response.

Long-term treatment of genetic disorders requires correction of deleterious variants in progenitor cells. It is widely accepted that basal cells can differentiate into many, or possibly all, respiratory epithelial cell types (20), a process recapitulated when primary airway cells are cultured at ALI (49, 50). In our study, basal cells, particularly suprabasal and cycling basal cells, were edited as indicated by the increase in *CFTR^+^* cells. It has been reported that basal cell transfection does not impact progenitor capacity (34) or drastically alter chromatin structure upon editing (45, 51). However, we observed a consistent reduction in the proportion of basal cells, both overall and among *CFTR^+^* cells, after editing. This decrease in basal cell fractions exacerbated differences from healthy ALI culture observed in our study and reported by others (18, 52). Likewise, the only secretory cell subtype to experience a decrease between unedited and edited samples was secretory club cells, which have progenitor capacity similar to that of basal cells. Whether this shift in progenitor cell populations could affect long-term reconstitution of the airway epithelium remains an important question that could be addressed by longitudinal study of airway epithelial changes in individuals treated with CFTR modulators. Additionally, future studies to track cell fate could determine whether the reduction of basal cells is due to enhanced differentiation into secretory cell subtypes.

Given the promising functional response and evidence of widespread editing across key epithelial cell types, we explored the potential of a clinically viable delivery method.

Our group had previously shown that polymeric NPs (PNPs) efficiently deliver RNA encoding GFP and Cre mRNA reporters to airway epithelial cells following systemic injection (30). Although correction in isogenic immortalized CFBE cells reached modest levels (5%–7% at the target nucleotide), CFTR function exceeded the 10% WT threshold. Interestingly, target base editing after PNP transfection was considerably higher in the primary cells (15%–27%) compared with immortalized isogenic cells. Despite lower editing frequencies, PNP-mediated delivery of the editing machinery achieved levels of CFTR function similar to those achieved by electroporation. One possible explanation is that the PNPs may be less toxic than electroporation, as previously reported (29). In addition, our in vitro results suggest that even low levels of CFTR correction may be sufficient for reversal of the CF phenotype (53).

Although modest correction may result in clinically relevant outcomes, it remains desirable to maximize the uptake of delivery vehicles such as NPs. In the case of lipid NPs (LNPs), vitronectin has been proposed as a key protein for in vivo targeting of lung SORT-LNPs (4, 54, 55). Thus, it was notable that vitronectin was the most abundant component of the lung-targeting PNP protein corona used here. However, vitronectin and albumin are the only proteins in the top 20 most abundant that both lung-targeting NP classes have in common, with physiological characteristics of the other abundant proteins varying greatly, suggesting that a targeting mechanism might lie in multiple contributing factors (56, 57). Notably, vitronectin was also the most abundant protein present on the protein corona of cardiac-targeting PNPs. Coating of PNPs encapsulating GFP mRNA with vitronectin did not improve transfection efficiency of either immortalized CFBE cells or fully differentiated primary airway cells. Our result is in contrast with that reported for lung-targeted SORT-LNPs, which may be due to the much higher abundance of vitronectin (~50% of protein) on lung-targeted SORT-LNPs, possibly suggesting a greater avidity of the LNP corona for vitronectin due to a more neutral surface charge (Supplemental Table 2) (4, 58). Despite the lack of vitronectin-enhanced uptake, up to 8% of basal cells were GFP^+^. Since differentiated airway cultures may be a reasonable proxy for airways in vivo, future studies should explore whether other PNP formulations with or without specific corona proteins substantially increase update in key cell types for CFTR correction.

The study has several limitations. A technical constraint of scRNA-seq is the limit of transcript detection. Modest increases in expression may translate into a disproportionate number of cells crossing the detection, thus accounting for a 4- to 7-fold increase in *CFTR* expression (43, 44). We report the reproducibility of this finding and propose explanations at the regulatory and cellular level, but we do not know whether this phenomenon is a consequence of correction of a splice site variant. Correction of other variants such as missense variants may have different effects (11, 12, 17, 18, 46). Differences in culture conditions, including days on ALI and choice of medium, influence cell type and lineage (23, 46, 59), as well as individual sample variability (18). The matched comparison used here reduced variability due to these effects. Studies with PNPs highlighted further improvement for both delivery of various cargoes and enhancement of base editor efficiency. Even though the amount of RNA delivered was scaled accordingly, allelic conversion rates never exceeded 27%, and although this level corrected *CFTR* function to clinical relevance in cell culture, it may be insufficient in vivo. Strategies to improve delivery could include optimizing PNP surface chemistry and enhancing editing efficiency with a more recently evolved and thus more accurate base editor to minimize potentially deleterious bystander effects (60). Finally, it remains unknown how well human primary cell culture translates to in vivo efficacy and why corrected primary cell subtype distributions migrated further from that seen in WT cultures.

In summary, these studies demonstrate that modest levels of editing may increase the level of *CFTR* in a disproportionate number of airway cells, thereby driving substantial improvement in CFTR-mediated transport to clinical relevance in CF. These studies provide key information about the cellular populations that undergo changes in expression when *CFTR* is successfully edited as well as in vitro evidence that informs translatability of PNP-mediated base editor gene therapy to potentially cure CF lung disease.

## Methods

### Sex as a biological variable

Sex was not considered as a biological variable, since all human samples used here were deidentified and thus not considered in the study design, as the objective was to establish technical feasibility rather than evaluate for biological variation.

### Polymer synthesis

Polymers were synthesized using previously reported protocols (61). Briefly, monomers bisphenol A glycerolate diacrylate (B7) and trimethylolpropane triacrylate (B8) were dissolved at 600 mg/mL in dimethylformamide reacted with side chain monomers [4-amino-1-butanol (S4), 4-(2-aminoethyl)morpholine (S90), and 1-dodecylamine (Sc12)] with 80:20 mol/mol c12:90 with stirring for 48 hours at 85°C to allow polymerization via stepwise Michael addition reactions. Monomers were reacted at an overall vinyl/amine ratio of 2.3 to allow acrylate-terminated polymers to form. Polymers were end-capped by further reaction with primary amine-containing E monomers (diethylenetriamine [E63]) at room temperature for 2 hours (200 mg/mL polymer and 0.3 M E monomer in tetrahydrofuran) and purified by 2 diethyl ether washes. Diethyl ether was decanted, and the sample was dried thoroughly under vacuum. The synthesized polymer, 7-90,C12-63 (PBAE-E63), was dissolved in dimethyl sulfoxide at 100 mg/mL and stored at –20°C with desiccant in single-use aliquots until used.

### Nanoparticle formulation

For in vitro studies, NPs were prepared by dissolving of polymer and mRNA at 1 μg/μL separately in 25 mM sodium acetate (NaAc; pH 5); then the 2 solutions were mixed at 1:1 volume ratio to allow for self-assembly at room temperature for 10 minutes.

### Cell culture

Primary HNE and HBE cells were propagated on a feeder layer of 3T3 mouse fibroblasts irradiated with 30 Gy before being isolated for transfection studies. To allow for expansion, cells were maintained in the presence of 10 mM reagent Y-27632 2HCl (ROCK inhibitor, Selleckchem). Passage number (P1, P2, P3, P4) reflects the time point of growth in a 6-well dish or T25 flask before transitioning to filters. Human bronchial epithelial (HBE) cells (CF: c.2988+1G>A/c.1521_1523delCTT; P3, WT: P3) were obtained through the Cystic Fibrosis Foundation Therapeutics Lab biobank. Passage number represents time since being thawed.

Immortalized bronchial epithelial cells from a person with CF (CFBE41o-) (62) with an integrated target for FlpIn recombinase (CF8Flp cells) (63) into which a *CFTR* expression minigene (EMG) containing 3120+1G>A (c.2988+1G*>*A) had been inserted were used for CFBE cell screens. The minigene contained all exons of *CFTR* and appropriately positioned flanking intron sequences from exons 14 to 18, as previously described (2). Cells were maintained in Gibco’s minimal essential medium with 10% FBS, 1% PS, and 200 mg/mL hygromycin B to maintain stable integration of the *CFTR* EMG. All cells were maintained at 37°C with 5% CO_2_.

### Transfection

#### Immortalized CFBE cells and primary HBE cells.

Immortalized and primary HBE CF cells were plated in either 24- or 6-well tissue culture plates and allowed to adhere. Once at approximately 80% confluence, cells were transfected with mRNA-encapsulating NPs. NPs were formulated following the in vitro transfection formulation described above; NP solution was added to 2 mL fresh medium, then incubated with cells for 2 hours before undergoing a change of medium. Forty-eight hours after HBE cell transfection, cells were moved to filters for differentiation. Cells remained in medium containing ROCK inhibitor until confluence was reached (about 1–2 days), at which point cells were moved to differentiation medium (no ROCK inhibitor). The next day, apical medium was removed, starting ALI culture. Primary cells were maintained on ALI culture for 14 days before short-circuit measurements and collection of genomic DNA (gDNA) and RNA. Cells transfected with GFP mRNA were collected 24 hours after transfection and stained with 7-Aminoactinomycin D (1:200 dilution) with transfection efficacy evaluated via flow cytometry (Attune NxT flow cytometer, Thermo Fisher Scientific) and analyzed using FlowJo software (FlowJo). The expression of GFP was quantified by normalization of the geometric mean fluorescence intensity (MFI) of each NP treatment to MFI of the untreated group. Gating strategies to identify GFP^+^ cell populations are provided in Supplemental Figures 10 and 11.

#### Primary HNE cells.

Base editor and sgRNA were delivered to nondifferentiated primary cells via electroporation. Base editor mRNA was at a concentration of 2 μg/μL, and synthetic sgRNA (Integrated DNA Technologies) was at a concentration of 100 μM. Editor and sgRNA were combined in a 3:1 volume ratio, and a total of 1 μL of RNA was used for each electroporation reaction. In the no sgRNA control, an equivalent volume of DEP-C H_2_O was substituted for synthetic sgRNA, and in the GFP control, eGFP mRNA with unmodified bases and CleanCap was used (TriLink Biotechnologies, catalog L-7601). Electroporation was performed using the Neon Transfection System (Invitrogen) with 10 μL Neon tips (Invitrogen, catalog MPK1025). Each electroporation reaction consisted of 1.5 × 10^5^ undifferentiated primary cells resuspended in 9 μL of buffer R (Invitrogen, catalog MPK1025) and combined with 1 μL of nucleic acid mix. Electroporation conditions were as previously described (2). After electroporation, cells were plated onto Snapwell filters (Costar, catalog 3801) coated with human collagen type IV (MilliporeSigma, C6745-1ML) in a 6-well plate seeded with irradiated mouse fibroblast cells (3T3). Two electroporation reactions were added to each filter. Fluorescence microscopy was performed 24 hours after electroporation to validate successful transfection of the GFP control. Cells were allowed to recover from electroporation in medium containing ROCK inhibitor until confluence was reached (about 5–10 days), at which point cells were moved to differentiation medium (no ROCK inhibitor). The next day, apical medium was removed, starting ALI culture. Cells were maintained on ALI culture for 21 days before short-circuit measurements and collection of gDNA and RNA.

#### Protein coating.

Primary HBE WT cells were grown on a feeder layer of irradiated 3T3 mouse fibroblast cells before being isolated and moved to filters, where they underwent the same timeline of differentiation as described above for 14–19 days before being transfected with NPs. Twenty-four hours after transfection, cells were harvested to create a single-cell suspension. Surface staining of cells with fluorescent antibodies was performed using the antibodies and dilutions listed in Supplemental Table 1 in FACS buffer for 30 minutes at 4°C, at which time cells were washed twice and resuspended in FACS buffer for analysis using an Attune NxT flow cytometer (Thermo Fisher Scientific). Data were analyzed using FlowJo software (FlowJo). Gating strategies to identify cell populations are provided in Supplemental Figure 11. Wells with at least 3,000 of each cell type were analyzed and reported from flow cytometry.

#### Genomic DNA extraction, PCR amplification, and sequencing.

For CFBE cells and primary airway cells, gDNA was collected directly from Snapwell filters after short-circuit measurements were taken. gDNA extraction was performed using the DNeasy Blood & Tissue Extraction Kit (QIAGEN, catalog 69504). After extraction, editing was assessed by PCR amplification of the relevant *CFTR* exon as previously described (2). For CFBE experiments, PCR was performed with KOD Hot Start Master Mix (MilliporeSigma). For primary cells, PCR was performed using HotStarTaq DNA polymerase (QIAGEN, catalog 203203). CFBE cells underwent linear/PCR sequencing performed by Plasmidsaurus using Oxford Nanopore technology with custom analysis and annotation. Primary HNE PCR products were purified before undergoing Sanger sequencing with editing rates at the target and bystander sites quantified with EditR (64). Primary HBE cell editing was assessed via MiSeq (see “High-throughput sequencing”).

### High-throughput sequencing

Targeted amplicons were generated using gene-specific primers with partial Illumina adapter overhangs (forward primer: 5-ACACTCTTTCCCTACACGACGCTCTTCCGATCTNNNN [gene-specific sequence]-3′; reverse primer: 5′-TGGAGTTCAGACGTGTGCTCTTCCGATCT [gene-specific sequence]-3′) and sequenced as previously described (65). Gene-specific primer sequences are listed in Supplemental Table 3. Extracted HBE cell gDNA (as described above) was used as a template to amplify the target site. Amplicons were indexed in a second PCR reaction and pooled for sequencing. Gel purification of the indexing PCR product on a 1% agarose gel was used to purify amplicons from primers. Purified amplicons were quantified using qPCR. 10% PhiX Sequencing Control v3 (Illumina) was added to the pooled amplicon library before running of the sample on a MiSeq Sequencing System (Illumina) to generate single-end 300 bp reads. Samples were demultiplexed using the index sequences, FASTQ files were generated, and alignments and editing quantification were conducted using CRISPResso2 (66).

### RNA analysis/cDNA synthesis

An input of 500 ng of RNA was used with the iScript cDNA synthesis kit (Bio-Rad). For immortalized cells, cDNA was diluted 1:10.

### Evaluation of CFTR channel function

#### Immortalized CFBE cells.

Cells were grown on filters until a transepithelial resistance greater than 200 ohms was reached (6–8 days). Filters were then mounted on Ussing chambers, and a chloride gradient was established using asymmetrical buffers as previously described (2). Short-circuit current measurements were taken using a multichannel voltage-current clamp amplifier (Physiologic Instruments) and the data acquisition program Acquire and Analyze. After equilibration, forskolin (10 mM) was added to the basolateral chamber to activate channel opening. The CFTR-specific inhibitor inh-172 was used (10 mM, apical chamber) to inhibit the channel. The drop in current after addition of inhibitor allowed for quantification of CFTR function (ΔI_sc_).

#### Primary cells.

CFTR function was assessed in primary cells by mounting of differentiated filters on Ussing chambers using a symmetrical buffer (126 mM NaCl, 25 mM NaHCO_3_, 5 mM KCl, 2.5 mM Na_2_HPO_4_, 1.8 mM CaCl_2_, 1 mM MgSO_4_, 10 mM dextrose). Buffer pH was maintained at 7.3–7.4 by continuous circulation with carbogen gas (95% O_2_/5% CO_2_), and temperature was maintained at 37°C. The apically located sodium epithelial channel (ENaC) was inactivated by addition of amiloride. A combination of forskolin and IBMX was added to the basolateral chamber to stimulate channel opening. Inh-172 was used to inhibit CFTR channel function and quantify ΔI_sc_.

### scRNA-seq

Library preparation was performed in the Cutting laboratory using 10x Genomics v3.1 3′ Library Preparation chemistry with a dual indexing system. Sequencing was performed by the Johns Hopkins Genetic Resources Core Facility via 2 × 100 cycles of paired-end sequencing using a NovaSeq 6000 Illumina sequencing machine. Analysis of scRNA-seq results was performed using 10x Genomics Cell Ranger 3.1.0 followed by the Seurat package (v4.2) in R created by the Satija laboratory (67). Subsetting was determined based on comparisons of the number of genes expressed, unique molecular identifier (UMI) count, and percentage of reads mapped to mitochondrial RNA. A log normalization with a scale factor of 10,000 was used. Cell type was assigned based on published transcriptional markers for each cell type: basal (6.4%; KRT5^+^, TP63^+^), cycling basal (2.2%; KRT5^+^, TOP2A^+^), suprabasal (9.1%; KRT5^+^, TP63^–^), secretory (32.0%; SCGB3A1^+^), secretory (club) (1.9%; SCGB1A1^+^), secretory (goblet) (3.0%; MUC5B^+^), secretory (club/goblet) (20.3%; SCGB1A1^+^, MUC5B^+^), mucous-multiciliated (10.9%; FOXJ1^+^, MUC5AC^+^), deuterosomal (1.5%; DEUP1^+^, FOXJ1^+^), ciliated (12.0%; DEUP1^–^, FOXJ1^+^), and ionocytes (0.7%; FOXI1^+^, *CFTR^+^*) (12, 13, 19, 21).

### Protein corona data collection and analysis

NPs formulated for in vivo use as described previously (30) were incubated in mouse blood serum (Bio-Rad) at 200 μL/mg for 1 hour and pelleted by centrifuge, then underwent preparation by Proteomics iST kit (PreOmics). Analysis was performed by liquid chromatography/mass spectrometry (LC-MS). Before MS analysis, samples were desalted using a 96-well plate filter (Orochem) packed with 1 mg of Oasis HLB C-18 resin (Waters). Tryptic peptides were analyzed on a Dionex RSLC Ultimate 300 (Thermo Fisher Scientific) coupled to an Orbitrap Exploris 480 mass spectrometer (Thermo Fisher Scientific). Peptides were separated using a 60-minute gradient from 4% to 30% buffer B (buffer A: 0.1% formic acid; buffer B: 80% acetonitrile plus 0.1% formic acid) at a flow rate of 300 nL/min. Briefly, for data-dependent acquisition MS, the full MS scan was set to 300–1,200 *m*/*z* in the Orbitrap with a resolution of 120,000 (at 200 *m*/*z*) and an automatic gain control (AGC) target of 5 × 10^5^. MS/MS was performed in the ion trap using the top speed mode (2 seconds), an AGC target of 1 × 10^4^, and an HCD collision energy of 35.

### Graphical illustrations

Graphical illustrations were created using BioRender (https://biorender.com/).

### Statistics

GraphPad Prism v10.1.1 (GraphPad Software) was used to perform all statistical analysis. Data are shown as mean ± SEM. Unless otherwise stated, the absence of statistical significance markings, where a test is stated to have been performed, signifies no statistical significance. Statistical significance is denoted as follows unless otherwise noted: *P < 0.05, **P < 0.01, ***P < 0.001, and ****P < 0.0001.

### Study approval

Primary HNE cells (c.2988+1G >A /c1438G>A; P3) were obtained by nasal brushing performed by Christian Merlo at Johns Hopkins Hospital under IRB no.00116966. Human bronchial epithelial (HBE) cells (CF: c.2988+1G >A/c.1521_1523delCTT; P3, WT: P3) were obtained through the CFF therapeutics lab biobank. All human subjects gave their informed written consent for inclusion before participation in the study.

### Data availability

Values for all data points in graphs are reported in the Supporting Data Values file. The RNA-seq data generated in this study were deposited in the Gene Expression Omnibus (GEO) database under accession number GSE324177.

## Author contributions

Conceptualization was contributed by EWK, ATJ, SYT, GAN, JJG, and GRC. Methodology was contributed by EWK, ATJ, ARP, ACE, and SYT. Investigation was contributed by EWK, ATJ, ARP, ACE, AIP, KLS, MJ, SRS, EY, and SYT. Visualization was contributed by EWK, GAN, and NS. Funding acquisition was contributed by EWK, SYT, NS, JJG, and GRC. Project administration was contributed by JJG and GRC. Supervision was contributed by JJG and GRC. Writing of the original draft was contributed by EWK, JJG, and GRC. Review and editing of the manuscript were contributed by EWK, JJG, and GRC.

## Conflict of interest

JJG reports a relationship with Dome Therapeutics that includes board membership, consulting or advisory, and equity or stocks. JJG reports a relationship with Cove Therapeutics that includes board membership, consulting or advisory, and equity or stocks. JJG reports a relationship with WyveRNA Therapeutics that includes board membership and equity or stocks. JJG reports a relationship with VasoRx that includes board membership and equity or stocks. GAN reports that he has filed patents US20230235309A1, US11795443B2, WO2025122725A1, and WO2026015609A1. Johns Hopkins University has filed a patent application, WO2024098053A1, based on technology discussed in this article with JJG, SYT, GRC, and EWK as co-inventors.

## Funding support

This work is the result of NIH funding, in whole or in part, and is subject to the NIH Public Access Policy. Through acceptance of this federal funding, the NIH has been given a right to make the work publicly available in PubMed Central.

- US National Institutes of Health (NIH) (R01EY031097 and P41EB028239 to JJG, R37CA246699 to SYT).
- Cystic Fibrosis Foundation (004319G222 to EWK, SYT, GRC, and JJG, CUTTIN20G0 to GRC, SHARMA23GO1 to NS, NEWBY23XX0 to GAN).
- The Spruance Foundation.
- Vertex Pharmaceuticals (Vertex Research Innovation Award to NS).

## Supplementary Material

Supplemental data

Supporting data values

## Version 1 05/08/2026

Electronic publication
